# S70. ABERRANT SALIENCE: A COMPARISON OF DIFFERENT MEASURES IN ANXIETY AND SCHIZOPHRENIA

**DOI:** 10.1093/schbul/sby018.857

**Published:** 2018-04-01

**Authors:** Suzanne Neumann, Richard Linscott

**Affiliations:** University of Otago

## Abstract

**Background:**

Aberrant salience is thought to play a role in the development of the symptoms of schizophrenia, but the hypothesis lacks consistent support. Previous research found no relationship in a population sample between two measures of aberrant salience: the self-report Aberrant Salience Inventory (ASI) and the computerised Salience Attribution Task (SAT), which measures implicit (behavioural) and explicit (self-report) aberrant and adaptive salience. We compared the ASI and SAT in individuals with schizophrenia, with anxiety, and with no mental disorder (unaffected).

**Methods:**

Individuals with schizophrenia (n = 30), anxiety (n = 33), or unaffected (n = 30) completed the ASI and the SAT.

**Results:**

ASI scores were higher in the schizophrenia group than anxiety (t(90) = 2.72, p < .01) and unaffected groups (t (90) = 5.29, p < .001) and higher in the anxiety than unaffected group (t(90) = 2.69, p < .01). SAT explicit adaptive salience scores were lower in the schizophrenia group than the anxiety (t(90) = -3.79, p < .001) and unaffected groups (t(90)= -3.86, p < .001). The schizophrenia group also had higher SAT implicit aberrant salience than the anxiety group (t(90) = 2.57, p < .05) but not the unaffected group (t(90) = 3.75, p = .08); there was no difference between anxiety and control groups (t(90) -0.76, p = .45). Group did not affect SAT explicit aberrant salience (F(2,91) = 0.47, p =.63) or implicit adaptive salience (F(90) = 0.62, p = .54). We found no correlation between the ASI and the SAT (all τ < .218, p > .05).

**Discussion:**

Higher ASI scores were associated with, but not unique to, schizophrenia. Reduced SAT explicit adaptive salience was associated with schizophrenia, while SAT implicit aberrant salience scores differed between psychopathologies. Consistent with previous findings, there was no relationship between the ASI and the SAT. The ASI is designed to measure a trait associated with schizophrenia. Conversely, the SAT implicit aberrant salience measures response latency to irrelevant stimuli. The lack of relationship between ASI and SAT may, therefore, be due to construct divergence.

